# Large Language Models in Nursing Education: Concept Analysis

**DOI:** 10.2196/77948

**Published:** 2025-08-22

**Authors:** Julia Harrington, Richard G Booth, Kimberley T Jackson

**Affiliations:** 1 Arthur Labatt Family School of Nursing, Western University, 1151 Richmond Street, London, N6A 3K7, Canada, 1 519-661-3395

**Keywords:** large language models, artificial intelligence, nursing education, undergraduate nursing education, graduate nursing education, concept analysis

## Abstract

**Background:**

Large language models (LLMs) are increasingly used in nursing education, yet their conceptual foundations remain abstract and underexplored. This concept analysis addresses the need for clarity by examining the relevance, meaning, contextual applications, and defining attributes of LLMs in nursing education, using Rodgers’ evolutionary method.

**Objective:**

This paper aims to explore the evolutionary concept of LLMs in nursing education by providing a concept analysis through a comprehensive review of the existing published literature.

**Methods:**

Rodgers’ evolutionary concept analysis method was used. PubMed, CINAHL, PsycINFO, Scopus, and Google Scholar were used to search for relevant publications. A total of 41 papers were included based on inclusion criteria that focused on studies published in English within the last 5 years to ensure relevance to the current use of LLMs exclusively in nursing education. Studies were excluded if they focused on clinical nursing applications, were not available in English, lacked full-text accessibility, or examined other artificial intelligence (AI) technologies unrelated to LLMs (eg, robotics).

**Results:**

As a result of this analysis, a proposed definition of LLMs in nursing education has been developed, describing them as accessible, personalized, innovative, and interactive tools that create revolutionary learning experiences, often leading to enhanced cognitive and skill development and improvement in learning and teaching quality.

**Conclusions:**

This concept analysis highlights LLMs’ transformative potential to enhance access to resources, support individualized learning, and augment nursing education. While promising, careful attention must be given to their limitations and ethical implications, ensuring their integration aligns with the values and goals of nursing education, particularly in specialized areas such as graduate nursing programs.

## Introduction

Over the last 5 years, there has been significant interest in the advancement of artificial intelligence (AI) in nursing education [1,2]. This includes a growing focus on AI-derived tools such as chatbots, adaptive learning systems, and AI-based patient simulations that offer opportunities to increase student engagement and motivation, improve learning outcomes, and support educators in their teaching efforts [3,4]. Recent discourse in nursing education has explored various intersections related to both educator and student engagement with AI, as related to aspects of the learning process [2,5], personalized instruction [6-8], interactive learning experiences [6,8,9], and enhanced access to diverse educational resources [6,9,10]. In addition to general discussion of AI in the learning process, the emergence of large language models (LLMs) has more recently gained popularity in the nursing education literature. LLMs are advanced types of AI systems trained on large volumes of text data to understand, generate, analyze, summarize, and predict additional content, simulating human-like interactions [11-13]. LLMs use deep learning, particularly transformer-based architectures, to process and produce coherent text responses based on user input [14].

The most widely known LLM is OpenAI’s Generative Pre-Trained Transformer, or ChatGPT. ChatGPT was launched in November of 2022 and quickly became a groundbreaking application, obtaining 1 million users within 5 days, and over 200 million active users worldwide [11,13,15,16]. As of spring 2025, ChatGPT appears to be the most widely used and studied LLM in nursing education [16]; regardless, other models such as Google Gemini and Microsoft Bing are also considered LLMs and belong to this category [11]. Following the release of ChatGPT, concerns arose within the academic community regarding academic integrity, academic dishonesty, and excessive use of technology by students, prompting the New York Department of Education to abruptly restrict access to the LLM over fears of academic integrity breaches [9,17-19]. While these concerns are valid, LLMs continue to be applied in nursing education to enhance clinical simulation scenarios, provide personalized support for assignments, assist students and educators in developing study materials, and improve learning by promoting interactive discussions, problem-solving, and critical thinking [2,5,6,12,16,19-21]. Despite the increasing usage of AI-powered tools in higher education [1,3], the concept of LLMs in nursing education remains conceptually abstract. This lack of clarity is due in part to inconsistent terminology across the literature, with terms like “chatbot,” “GenAI,” and “LLMs” often used interchangeably without a clear distinction. Furthermore, there is a scarcity of formal definitions that delineate the role and scope of LLMs within nursing pedagogy. This conceptual ambiguity is further compounded by the evolving nature of the AI-powered technology itself and the variability in how learning outcomes are defined and assessed across educational settings [22]. Subsequently, the purpose of this paper is to explore and clarify the concept of LLMs in nursing education using Rodgers’ [23] evolutionary concept analysis method. The analysis will examine and present the surrogate terms, defining attributes, antecedents, consequences, empirical referents, and a model case to further situate LLMs within the broader educational and technological landscape. As a result of this concept analysis, a definition of LLMs in nursing education will be developed and presented. Due to the limited clarity related to the theoretical foundations or practical applications of LLMs in nursing education, this concept analysis is important toward advancing understanding and guiding future research and practice of this emerging concept.

## Methods

### Design

Rodgers’ [23] evolutionary method of concept analysis was chosen to analyze LLMs in nursing education because it accommodates the dynamic, context-dependent nature of emerging technologies, enabling an exploration of how the concept evolves over time and its significance in education [23]. Conducting a concept analysis using the Rodgers [23] method involves identifying the concept, its surrogate terms, and selecting suitable data collection methods; gathering data to determine attributes, antecedents, and consequences; and illustrating the concept with examples while identifying areas for further development [24]. Rodgers’ [23] evolutionary concept analysis consists of several phases that are flexible and iterative, rather than following steps in a linear progression. The methodology outlined by Duffy et al [24] in their concept analysis of relational connection in telehealth practice was used to guide the methods of this analysis, as it offers a clear and systematic approach to examining evolving concepts within nursing practice in the context of emerging technologies.

### Data Collection

A comprehensive search was conducted by the lead author between January and March 2025 across 5 databases: PubMed, CINAHL, PsycINFO, Scopus, and Google Scholar. Keywords included “large language models,” “LLMs,” “language model,” “genAI,” “conversational AI,” “ChatGPT,” “chatbot,” “artificial intelligence,” “AI,” “nurs* education,” “undergraduate nurs* education,” and “graduate nurs* education.” Boolean operators and truncation were used to optimize search sensitivity. Inclusion criteria focused on studies published in English in the last 5 years to ensure relevancy of the use of LLMs exclusively in nursing education. Exclusion criteria eliminated studies that focused on LLMs in clinical nursing settings, studies not published in English, studies lacking full-text accessibility, and studies that focused on other forms of AI technology unrelated to LLMs (eg, robotics). Antecedents of the concept were identified primarily from included articles; however, supplemental literature outside the inclusion criteria was also referenced. This approach was necessary, as not all included articles explicitly discussed antecedents of LLMs in nursing education. Titles were screened for relevance by the lead author, followed by abstract reviews for eligibility. Articles meeting the inclusion criteria were read in their entirety, and those that did not address the concept of LLMs in nursing education were excluded from the analysis. Reference lists of included articles were used to locate additional sources. In total, 41 articles were included; 36 met inclusion criteria directly, and 5 additional sources were included to support the discussion of antecedents. While the Elo and Kyngäs [25] method of inductive content analysis was not formally adopted, the thematic analysis conducted in this study closely aligns with its principles, including category development and abstraction to identify key attributes, antecedents, and consequences of LLMs in nursing education [25]. A total of 9 studies used qualitative methodology [9,12,17,26-31], 11 used quantitative designs [6,7,32-40], 1 used a mixed methods approach [41], 2 were educational evaluations or frameworks [16,42], 6 were narrative or discussion papers [11,19,43-46], and 7 were systematic, literature, scoping, or rapid reviews [2,5,8,18,20,47,48]. Among the 5 articles included for the discussion of antecedents, 1 was a policy report [49], 1 a professional standards document [50], 1 a conceptual framework [51], 1 a discussion paper [52], and one a literature review [53].

## Results

The second step in Rodgers’ [23] method of analysis involves identifying surrogate terms of the concept [20]. Currently, the nursing education literature uses a variety of definitions or terms to describe the concept of LLMs. Terms used to refer to the concept of LLMs in nursing education included AI-based LLMs [42]; AI model [31]; AI tool, system, or platform [6,9,31,38,42]; AI application [6,7,17,28,42]; technological support for learning [42]; generative AI [5-7,17,28,31,32,35,38,41,46]; large language technology [33]; text-generated language models [6]; natural language processing model or program [8,28]; ChatGPT chatbot [33]; generative pretrained transformers [7]; AI-powered chatbot [2,5,27,34,41]; automated conversational agents [2]; conversational AI [20]; software [31]; educational tool [7]; and learning assistance tool [39]. The included papers were reviewed to determine the attributes, antecedents, and consequences of LLMs in nursing education. Table 1 demonstrates a summary of the relationship between the antecedents, attributes, and consequences of the concept.

**Table 1.** Relationship between antecedents, attributes, and consequences of large language models (LLMs) in nursing education.

Antecedents	Attributes	Consequences
Technological infrastructure (eg, a device capable of running the LLM^a^ and internet connection)	Accessibility—easy to use across platforms; supports diverse users	Enhanced cognitive skills and learning development (eg, improvements in critical thinking, clinical reasoning, decision-making, and learner confidence) Linked to improved efficiency, creativity, self-efficacy, personal growth, communication, and problem-solving skills
Technological advancements (eg, transformer architectures)	Personalization—tailors content to user’s learning needs, preferences, and goals	Increased quality and accessibility of education: supporting educators in creating exam questions and simulation scenarios Immediate access to information facilitates engagement, improving their learning experience Supports global collaboration in nursing education
Data availability: LLMs require and analyze extensive datasets	Interactivity—real-time, dynamic responses; supports active learning and simulation integration	Ethical and academic integrity concerns: raise questions around intellectual property and plagiarism
Digital transformation in education	Revolutionary nature—represents a paradigm shift in teaching; supports innovation in clinical and theoretical areas	Practical limitations: may produce inaccurate information, weaken critical thinking and decision-making
Shift toward competency-based, outcomes-focused education	—^b^	Potential for bias and over-reliance: may perpetuate biases in nursing education
Acceptance of AI^c^ among students and faculty; social influence and motivation	—	—

a LLM: large language model.

b Not available.

c AI: artificial intelligence.

### Attributes

#### Overview

When the attributes of a concept are unclear, practitioners may struggle to apply it effectively in essential tasks [23]. To assist with this, Rodgers’ [23] fourth method of analysis includes the definition of attributes of interest. Through this process, recurring themes and key descriptors used to characterize LLMs in educational settings were identified, highlighting their impact and functionality. A total of 4 attributes were identified as the most consistently cited features, reflecting how LLMs adapt to individual needs, promote engagement, and transform nursing education. These identified attributes are (1) accessibility, (2) personalization, (3) interactivity, and (4) revolutionary nature.

#### Accessibility

A key defining attribute of LLMs in nursing education is their accessibility, a characteristic that has been highlighted across the literature as essential to their effectiveness in diverse educational settings, including undergraduate and graduate nursing programs [2,5,6,9,17,42,46]. The accessibility of LLMs enables students and educators to leverage LLMs without the barriers of cost or technical expertise [40]. LLMs are further distinguished by their accessibility on various devices, such as smartphones and laptops, allowing users to access their capabilities at any given moment, thus enhancing convenience and usability [43]. In addition, LLMs can significantly improve accessibility for non-English speakers and students with writing disabilities, as these individuals often face challenges with traditional methods of communication [42]. LLMs in nursing education offer new avenues for expressing ideas and demonstrating knowledge, breaking down language barriers [42]. Throughout the included literature, LLMs in nursing education were consistently highlighted for their accessibility, providing rapid and opportune access to vast amounts of information, allowing students and educators to efficiently retrieve and synthesize knowledge without the need to sift through extensive manuals or academic texts [16,18,45].

#### Personalization

Personalization was identified as a defining attribute of LLMs in nursing education, reflecting their innate ability to generate individualized learning experiences for users, adapting to one’s unique needs, abilities, preferences, goals, and learning styles [18,26,36]. Numerous studies highlight LLMs’ potential to improve nursing education via personalized interactions and responses when describing LLMs in nursing education [6,12,18,29,34,36]. ChatGPT in particular has been recognized for its ability to adapt to user preferences, enabling nursing students to customize its use in ways to achieve their individual learning goals [17]. The integration of LLMs offers a more personalized approach to nursing education, with students benefiting from individualized learning experiences [12]. Parker et al [29] emphasize ChatGPT’s role as a “powerful self-learning” tool, highlighting its ability to engage with nursing students in personalized interactions. The personalization of LLMs extends to practical application in nursing education, as they can generate personalized study guides, act as an online tutor, and create tailored learning plans based on students’ abilities to improve learning efficiency [18]. By individualizing learning opportunities, LLMs enhance learning efficiency, improve academic performance, and reinforce the value of personalization in nursing education [6,36].

#### Interactivity

Interactivity was identified as a defining attribute of LLMs in nursing education, particularly due to their ability to facilitate interactive learning and provide real-time feedback in response to user inputs [36]. LLM’s ability to contextualize user queries promotes a bidirectional exchange, which is considered crucial in nursing education for reinforcing complex concepts [36]. Due to their interactivity, nursing educators are integrating LLMs into diverse settings, such as simulation-based education, to enhance the overall learning experience [46]. These simulations provide students with realistic patient interactions, helping them improve communication, history-taking, and clinical decision-making skills in a controlled environment [20,48]. Beyond simulations, LLMs are reshaping learning experiences by increasing student engagement and interaction [6,20]. This attribute distinguishes LLMs from other AI technologies, such as rule-based systems (eg, decision support systems), which lack the ability to adapt to user input in real time or facilitate an interactive educational experience [54]. LLMs’ interactive capabilities set them apart from traditional chatbots by enabling more dynamic and meaningful conversations that encourage interactivity and adaptation in learning [6,9]. LLMs’ advanced capabilities allow for deeper engagement through interactive learning experiences and LLM-generated study guides, which can assist students in developing critical thinking skills and gaining a more comprehensive understanding of course material [46].

#### Revolutionary Nature

The revolutionary nature of LLMs was identified as an attribute in nursing education due to their transformative impact across multiple dimensions of teaching, learning, and professional preparation [27,34,40,44,45]. LLMs in nursing education represent a paradigm shift, redefining traditional teaching methodologies by moving beyond passive didactic instruction toward dynamic, interactive, and technology-driven learning experiences [18,36]. Their revolutionary nature is reflected in their expansive potential to support skill development, clinical training, writing proficiency, research, and creativity, aligning with findings from non-nursing disciplines that position LLMs as a critical innovation in modern education [2,38]. Maykut et al [27] argue that the integration of LLMs into nursing education embodies the “fourth technological revolution,” signifying a shift in how knowledge is digitalized, disseminated, and applied. ChatGPT stands out as a particularly influential LLM, offering unique opportunities to extend learning beyond the classroom by fostering knowledge acquisition, analysis, and application. Given its profound influence on student learning experiences, ChatGPT has emerged as a critical area of nursing research, necessitating ongoing evaluation of its effects on the learning process and overall academic performance [6]. As LLMs progress, their revolutionary nature will influence nursing education to evolve, bridging gaps in clinical preparedness, fostering digital literacy, and redefining the future of nursing education [27,45].

### Antecedents

Antecedents are the events or phenomena that need to be present before LLM use in nursing education [23,24]. In this analysis, antecedents were identified as (1) technological infrastructure; (2) technological advancements; (3) data availability; (4) digital transformation in education; (5) the shift in nursing education toward competency-based, outcomes-focused learning; and (6) AI acceptance in nursing education. The primary antecedent of LLMs in nursing education is technological infrastructure, such as a device capable of running the LLM and an internet connection, ensuring accessibility for most educational settings [53]. Technological advancements, such as the development of transformer architectures (eg, GPT) are an antecedent as these developments allow LLMs to handle complex language tasks such as simulating clinical scenarios and providing instant feedback, making them valuable tools in nursing education [11,30,53].

Another antecedent is data availability; LLMs can assist students and educators by analyzing extensive datasets to generate personalized recommendations, customized learning experiences, immediate answers to clinical questions, and support clinical decision-making [55]. To enhance LLMs’ effectiveness in diverse educational and practice settings, training LLMs with local datasets is essential [47]. This approach ensures response accuracy by aligning the model with the institution’s specific practices while ensuring adherence to local policies and regulatory standards [47]. Concurrently, the “digital transformation of education,” with the widespread adoption of learning management systems and internet-based platforms, acts as an antecedent to the integration of LLMs into nursing education [27,49].

The shift in nursing education toward competency-based, outcomes-focused learning acts as a key antecedent for LLMs in nursing education. This change is reflected prominently in guidelines such as the American Association of Colleges of Nursing recommended competencies [16,50] and the Canadian Association of Schools of Nursing National Nursing Education Framework [51]. However, it should be noted that this pedagogical emphasis on competency-based frameworks primarily represents a Western perspective. Not all literature reviewed explicitly addressed such an educational approach, and global variations in nursing curricula must be acknowledged. Despite these contextual differences, the increasing global focus on synthesizing skills and knowledge, alongside the integration of emerging technologies such as AI, aligns closely with the capabilities of LLMs to enhance competency development and clinical decision-making across diverse educational settings [16,50,51].

The growing acceptance of AI in nursing education serves as a crucial antecedent to the integration of LLMs, shaping both educators’ and students’ attitudes toward their adoption [52]. LLMs such as ChatGPT have generative and analytical capabilities that can reduce educators’ workload [18]. From a student perspective, motivation to use LLMs is primarily driven by perceived value, including convenience, efficiency, and enhanced quality of their work [9]. Furthermore, subjective norms play a pivotal role in shaping students’ decisions to adopt LLMs, as peer influence and social media exposure act as key determinants of new technology adoption [9]. The phenomenon of fear of missing out (“FOMO”) further accelerates LLM use in nursing education as students may perceive themselves as missing out if they do not use AI tools that hold potential to improve their study performance [9]. In addition, the increasing integration of mobile phones, social media, and digital communication platforms is expected to drive the prevalence of LLM usage in nursing education [9]. LLMs’ ability to enhance learning engagement and satisfaction further reinforces the growing shift toward AI acceptance in nursing education, emphasizing its role as a key antecedent in the adoption of LLMs [2]. However, organizational pressures, including restrictions from academic institutions, educators, and hospitals, may act as barriers to LLM adoption. Ma et al [9] found that some nursing students reported a diminished motivation to use LLMs due to prohibitions on use in specific contexts, whereas others experienced heightened curiosity due to the “forbidden fruit effect,” where restrictions increased interest and engagement.

### Consequences

Consequences are what occurs after or as a result of the concept being applied [56]. The included literature demonstrates several positive outcomes of LLMs in nursing education. The positive consequences have been grouped into two broad categories: (1) enhanced cognitive skills and learning development and (2) increased quality and accessibility of education. Alongside these consequences, some of the included literature raises concerns about possible negative consequences. While these concerns are emerging rather than empirically established, they warrant consideration as LLMs become more integrated into educational settings.

The integration of LLMs into nursing education has led to enhanced cognitive skills, including improvements in critical thinking, clinical reasoning, decision-making, and learner confidence [2,5,6,11,19,20,32,34,39,41,47,57]. Furthermore, the use of LLMs in nursing education has been linked to improved learning capabilities, efficiency, creativity, self-efficacy, personal growth, communication, and problem-solving skills [6,18,19,32,34,38,39,42]. Studies have found that LLMs provide personalized support, enabling students to grasp complex concepts more effectively and engage in self-directed learning and practice problem-solving in real time, maximizing their educational outcomes and professional skills [2,6,18-20,32,34,39,42]. While still emerging in practice, LLMs such as ChatGPT are increasingly used in nursing education, supporting educators in item generation and content preparation while expanding learning opportunities for students [7]. By assisting faculty members in generating simulation scenarios and exam questions, LLMs can reduce educators’ workload, allowing them to focus on critical aspects of teaching, directly improving the quality of nursing education provided [5,7]. LLMs’ ability to provide immediate access to information and instant feedback facilitates engagement, allowing learners to study efficiently based on their individual needs while improving their overall learning experience [17,19,41]. Furthermore, LLMs can enhance educational accessibility by supporting remote education for students in rural and underserved areas [5]. When integrated into nursing education, LLMs can facilitate global collaboration between nurse academics and students while blending traditional and digital learning approaches [5].

However, alongside these positive consequences, the literature also identifies several potential negative consequences associated with LLM use in nursing education. A frequently cited concern involves issues related to ethics and academic integrity. LLMs may generate content that lacks proper attribution, misrepresents sources, or closely resembles copyrighted materials, raising questions about plagiarism and intellectual property [9,18,19,29,30,34,39,42,47,57]. Another concern centers on the practical limitations of LLMs, particularly regarding the accuracy and currency of their output. Since LLMs depend on historical data and may not always reflect the latest clinical guidelines, they can inadvertently produce outdated or inaccurate information, which can pose a significant barrier to their safe application in practice-based education [5,13,18,34]. The potential for bias and over-reliance is also a critical area of concern. LLMs are trained on large datasets, often with limited transparency, which may embed or perpetuate existing biases in nursing education [18,19,30,33,57]. By providing immediate, automated responses, LLMs may weaken students’ critical thinking, problem-solving skills, learner creativity, and ability to engage in independent analysis and decision-making [6,33]. Furthermore, the ease of access to LLMs could lead to automation bias, where students place undue trust in LLM-generated responses without critically evaluating their accuracy or applicability [18]. Assessing the true extent of these negative consequences is challenging, as students may underreport their dependence on LLMs, further complicating efforts to address this issue [9].

### Empirical Referents

The sixth step of Rodgers’ [23] evolutionary concept analysis requires the identification of concepts that are related to the concept of interest [23]. Instead, empirical referents or ways in which we see LLMs being used in nursing education will be discussed. This adaptation allows the concept analysis to provide more actionable insights in the field of nursing where practical application is critical [58]. This adjustment aligns with Rodgers’ [23] framework as the method is inherently flexible and encourages adaptation to the concept’s dynamic nature [23,59].

In nursing education, LLMs are used in three primary ways: (1) to support students in assignments and learning activities, (2) to assist faculty in developing and evaluating exam questions, and (3) to evaluate the performance of LLMs on nursing licensing exams. In undergraduate nursing education, LLMs are commonly used to enhance learning experiences by providing tutoring, generating explanations for complex nursing concepts, and assisting with academic writing [2]. For example, Tseng et al [38] found that nursing students frequently used ChatGPT for case report writing and academic support, although some struggled to connect nursing problems to individualized care plans due to overreliance on LLM-generated content. Similarly, Gonzalez-Garcia et al [6] observed that students using ChatGPT in coursework demonstrated improved academic performance, with women specifically reporting benefits in completing academic tasks. Some institutions have adopted structured LLM frameworks, such as OpenAI’s CIDI (Context, Instructions, Details, Input) model, to facilitate decision-making through guided question strategies, differentiating it from more flexible systems like ChatGPT [32]. In addition, LLMs are being increasingly used to support simulation-based learning by generating clinical scenarios that help students refine critical thinking and problem-solving skills [5]. In graduate nursing education, LLMs are being used as research and programming support tools, such as assisting PhD students with data analysis [41] and guiding students on AI integration in coursework [16]. Furthermore, LLM-generated clinical data has been piloted in Doctor of Nursing Practice courses to develop competency and clinical judgment [30].

Faculty in nursing education have incorporated LLMs into the development and evaluation of exam questions to streamline item generation, assessment feedback, and case-based learning [7,29,33]. Cox et al [33] explored how ChatGPT can be used to generate National Council Licensure Examination for Registered Nurses (NCLEX-RN) examination questions, comparing LLM-generated items to those created by nursing educators. When in combination with human expertise, researchers found this LLM can assist faculty with item generation to help prepare nursing students for the NCLEX-RN examination [33]. Similarly, Parker et al [29] demonstrated the usability of LLMs in nursing education using ChatGPT as an automated writing evaluation (AWE) tool. Educators can enhance the feedback process without adding to their workload, while students can benefit from personalized feedback by integrating the AWE capabilities of this LLM into the writing process [29]. This integration allows for efficient, individualized support that accelerates learning and improves writing quality [29]. In addition, Higashitsuji et al [7] investigated the use of ChatGPT in case-based learning, where the faculty used the model to generate case scenarios significantly reducing the time required for case creation.

Researchers have assessed LLMs’ ability to answer nursing licensure examination questions, evaluating their potential role in exam preparation and competency testing. Wu et al [39] analyzed the performance of LLMs on the National Nursing Licensure Examination (NNLE) in China and the NCLEX-RN in the United States and Canada. Through inputting exam questions to various LLMs, including OpenAI’s GPT-4, GPT-3.5, and Google’s PaLM, researchers compared their accuracy in responding to nursing-related content. This process allows educators and researchers to assess how well LLMs interpret and apply nursing knowledge under standardized testing conditions. The ways in which LLMs are being used in nursing education highlight their potential to serve as a valuable resource, aiding in the preparation for licensure exams and enhancing clinical decision-making skills [37,39].

### Model Case

The model case, a significant component of Rodgers’ [23] evolutionary concept analysis, is best identified through a real-world example rather than being constructed [23]. Including a model case that illustrates everyday instances of the relevant attributes helps clarify and strengthen the understanding of the concept [60]. The model case has been adapted from a real-world use case of ChatGPT described by Chang et al [32]; however, the specific identification of antecedents, attributes, and consequences has been applied based on the findings of this concept analysis.

In a third-year undergraduate nursing course focusing on health education design, students are tasked with creating a dietary education sheet tailored for pregnant women in their first trimester. This course is offered within a digitally progressive institution that has invested in advanced technological infrastructure and embraces AI integration as part of a broader educational transformation. Recent advancements in AI tools, the widespread availability of data, and the institutional shift toward competency-based, outcomes-focused learning have created an environment conducive to innovation. This setting reflects the growing acceptance of AI in nursing education and supporting the seamless adoption of LLMs to enhance teaching and learning practices. Recognizing the limitations of traditional teaching methods, the course integrates a revolutionary approach using an openly accessible LLM (ChatGPT). At the beginning of the course, students log into the LLM on their institution-provided laptops, which are part of a robust technological infrastructure supporting advanced learning tools. The LLM provides personalized guidance, addressing individual inquiries and offering tailored resources to meet each student’s learning needs. The LLMs’ adaptive responses clarify the student’s doubts and introduce critical thinking prompts, challenging them to justify their choices based on the evidence. Throughout the course, the LLM facilitates ideation by allowing students to brainstorm solutions collaboratively. The interactive nature of the LLM transforms the learning experience into a dynamic, inquiry-based process. Students practice designing educational materials iteratively, receiving immediate feedback from the LLM, which they further refine in collaboration with their peers and instructors. This innovative, interactive framework fosters an environment where students can develop critical thinking skills and deepen their understanding of patient education. The students demonstrate enhanced cognitive abilities and learning development as evidenced by their ability to critically analyze complex health education scenarios and create effective solutions. Educators devote more time to mentoring and addressing individual learner needs using the LLM in this nursing course.

## Discussion

### Principal Findings

Although the use of LLMs in nursing education is becoming increasingly prevalent, there has been significant oversight in clarifying their theoretical foundations and practical applications. Applying Rodgers’ [23] method of concept analysis provides a deeper understanding of LLMs’ unique qualities and applications within the context of nursing education. Based on the findings of this analysis, LLMs in nursing education can be defined as accessible, personalized, innovative, and interactive tools that create revolutionary learning experiences, often contributing to enhanced cognitive and skill development, as well as improvements in learning and teaching quality. Key characteristics frequently associated with LLMs in nursing education include accessibility, personalization, interactivity, and their revolutionary nature. One of the key findings of this concept analysis is the variation in terminology used to describe LLMs in nursing education. Various studies use different terms to define the use of LLMs in nursing educational contexts, making it challenging to form a unified understanding of the concept. Although more recent studies appear to be aligning around the term LLMs [5,8,9,31,35,38,61], there is still a lack of consistency in definitions. The term LLMs is continually evolving as the technology and its applications in nursing education develop [62]. It is important to establish a standardized terminology as these have been shown to have positive effects on clinical practice, enrich nurses’ knowledge, and shift attitudes toward education and guidance [63]. Future research should prioritize refining and standardizing definitions to ensure clarity and facilitate more precise discourse in the field of nursing education.

As research on LLMs in nursing education continues to evolve, this analysis highlights an opportunity to explore their potential applications in nursing practice. The role of LLMs in clinical decision-making, patient care, and interprofessional collaboration remains underexplored, limiting our understanding of how LLMs may support or hinder nursing practice [9]. Similarly, Ma et al [9] suggest that nursing students, particularly those engaged in clinical practice, rely heavily on knowledge resources to ensure patient safety and quality care. This further emphasizes the need to examine how LLMs may influence these critical aspects of nursing practice. In addition, the outcomes of using LLMs in nursing education are not fully understood. While some studies report positive outcomes, such as enhanced student engagement, critical thinking, and problem-solving skills, others present a more balanced perspective, acknowledging both benefits and challenges, including ethical considerations, bias, and the risk of overreliance [2,5,6,9,11,18-20,32,34,35,38,39,41,42,47,57]. Given these are varied perspectives, further research can help build a clearer understanding of how LLMs influence nursing education and practice and whether their integration translates into meaningful improvements in nursing competency. One emerging ethical concern is the potential for gender bias in LLM outputs, which has been well-documented in recent research [64-66]. Gender bias can manifest in subtle ways, such as the reinforcement of stereotypically masculine traits in leadership examples or the undervaluing of female-associated attributes [64]. LLMs trained on gender-imbalanced datasets may unintentionally reproduce societal stereotypes, which could negatively affect how students perceive themselves and their professional roles [64].

As LLMs continue to be integrated into nursing education, efforts to establish structured guidelines on their implementation are still evolving. Some theoretical models have been proposed to help educators and students navigate the use of LLMs, yet their adoption varies across institutions. For instance, the IDEE framework created by Su and Yang [67], while not specific to nursing education, offers a structured approach for integrating AI into general education settings by identifying desired outcomes, determining appropriate levels of automation, ensuring ethical considerations, and evaluating effectiveness [17]. The IDEE framework offers a potential roadmap for educators looking to integrate LLMs and generative AI into their teaching practices. Similarly, Abujaber et al [43] applied a strengths, weaknesses, opportunities, and threats (SWOT) analysis to assess the integration of ChatGPT into nursing education, identifying both the advantages and challenges of its use. Abujaber and colleagues’ [43] findings provide recommendations for educators, policymakers, and students to maximize benefits while mitigating risks. In addition, Blomquist et al [42] proposed a model that leverages existing decision-making and delegation frameworks to guide ethical LLM use in nursing practice. This approach suggests that nursing faculty and students could adopt structured decision-making principles to determine when and how LLMs should be applied. Furthermore, Goktas et al [68] developed specific guidelines for using OpenAI’s GPT-4.0 in nursing education, using the prompt learning method to optimize student engagement and learning outcomes. Collectively, these frameworks highlight the diverse strategies being explored to ensure the effective and ethical integration of LLMs into nursing education, reinforcing the need for continued refinement and adaptation as these technologies evolve.

### Limitations

Rodgers’ [23] methodology of concept analysis, while effective in examining the attributes of a concept such as LLMs, has certain limitations when applied to contemporary technological phenomena in nursing education. First, the review was shaped largely by peer-reviewed studies from Western-based contexts, which may limit the transferability of findings to global or culturally diverse educational environments. This Western-centric lens risks overlooking region-specific challenges, values, or innovations in LLM use. In addition, the search strategy may have constrained the conceptual scope; the reliance on specific terms such as “LLMs” and “ChatGPT” likely excluded literature that described similar technologies using alternative terminology. As a result, relevant studies may have been unintentionally omitted. Although the thematic analysis in this analysis was informed by the principles of Elo and Kyngäs’ [25] inductive content analysis, the absence of a formally structured analytic framework may limit the methodological rigor and replicability of category development and abstraction processes. Furthermore, while the negative consequences discussed are largely potential concerns rather than documented outcomes of LLM use in nursing education, they were included to reflect the emerging discourse and to encourage proactive consideration of possible risks as the technology continues to evolve. Finally, while the Rodgers [23] method emphasizes the evolutionary nature of concepts, it does not inherently accommodate the rapid pace and interdisciplinary complexity of technological innovation. As a result, a more inclusive, adaptive, and globally comprehensive approach may be necessary for future concept analyses involving emerging digital technologies in nursing education.

### Conclusions

The concept of LLMs in nursing education represents a dynamic intersection of technology and pedagogy, marked by rapid evolution and untapped potential. This analysis reveals that while LLMs are reshaping learning through personalized, interactive, accessible, and revolutionary experiences, their meaning will continue to evolve alongside educational practices and technological advances. In addition, LLMs should be understood as adaptable tools whose value depends on thoughtful application and continuous evaluation. Moving forward, the nursing education community must foster a shared understanding of LLMs—one that balances innovation with caution, and usage with responsibility. As these technologies become increasingly embedded in educational and clinical contexts, future efforts should prioritize developing evidence-based guidelines, addressing conceptual ambiguities, and cultivating digital fluency among both learners and educators. Only then can LLMs be fully harnessed to enhance not just how nurses learn, but how they think, decide, and care.

## Abbreviations:

AI: artificial intelligence
LLM: large language model
NCLEX-RN: National Council Licensure Examination for Registered Nurses
